# Use of digital devices, family functioning, and language development in preschool children: a cross-sectional study

**DOI:** 10.1590/2317-1782/20232023125en

**Published:** 2024-03-18

**Authors:** Maria Inês Figueiras Gomes, Marisa Lobo Lousada, Daniela Maria Pias de Figueiredo

**Affiliations:** 1 Escola Superior de Saúde, Universidade de Aveiro - ESSUA, Aveiro, Portugal.; 2 Centro de Investigação em Tecnologias e Serviços e Saúde - CINTESIS, Universidade de Aveiro, Aveiro, Portugal.

**Keywords:** Language Development, Child Development, Preschool Age, Digital Technology, Family Dynamic

## Abstract

**Purpose:**

This study aimed to analyse the relationship between the use of digital devices, family function, and language development in preschool children.

**Methods:**

This cross-sectional, descriptive-correlational study included a sample of 93 parent-child dyads. The children were of an average age of 57.01 ± 9.95 months, and the majority were female. The data collection instruments included a questionnaire on the use of digital devices, the Portuguese version of the Family Flexibility and Cohesion Evaluation Scale - Version IV (FACES-IV), and a Preschool Language Test (TL-ALPE).

**Results:**

The findings showed a greater tendency of children to use smartphones, tablets, and television for 0-3 hours daily. The analysis of the responses on the FACES-IV and TL-ALPE instruments showed that most of the participating families were of the balanced type and that most children had normal language development. Statistically significant relationships were found between the FACES-IV subscales and TL-ALPE subtests, FACES-IV subscales and the use of digital devices, and the use of digital devices and TL-ALPE subtests. Notably, children in more balanced family functioning scored higher on TL-ALPE tests, and the time spent using digital devices may compromise language development.

**Conclusion:**

This study highlights the impact of digital device use and the role of family functioning on children’s language development, suggesting that moderate digital device use and balanced family functioning are facilitating factors for good language development.

## INTRODUCTION

Adequate language development is one of the main factors for harmonious children’s development in all domains, whether from a social, relational, or formal learning point of view. It is a progressive, differential, and specific process for each child^(1,2)^. Language is acquired naturally and spontaneously and manifests itself through the acquisition of a language. For this to happen, the child must be exposed to that language^(3)^. Language development follows a sequential order of acquisition and developmental milestones that occur at approximately the same age in all children. However, throughout language development, there are individual differences in terms of the acquisition process and the quality and speed of the acquisition^(3)^.

As children acquire the language used in the community in which they grow up, their linguistic behaviour reflects the differences and specificities between the system and their surroundings^(2)^. Consequently, language development not only depends on the innate biological conditions of each individual but is also influenced by the environmental factors present in the children’s environment, such as the family or school^(4)^. Regarding biological conditions, particularly genetic factors, some authors indicate that a history of language disorders in the family directly influences the child, so it is common for children with a language disorder to have at least one relative who also had a similar alteration in childhood. Studies have also indicated that language disorders are more prevalent in male children, suggesting that the sex of the child is also a relevant genetic factor^(4)^. Further, the child’s environment is fundamental to the acquisition and development of language, since without this influence, genetic competencies for language acquisition will not be developed. The environment therefore plays a crucial role in the process of language acquisition and development, since children develop progressively and naturally through exposure to the language of the community in which they live, according to their individual pace of development^(4)^.

A prominent environmental factor that influences language development is the family, including the parenting styles adopted, the family socio-economic level, the parents’ education, and the presence or absence of siblings, among others^(4)^. More stimulating environments have an essential influence on brain organisation, providing richer interaction experiences that enable cognitive, functional, social, emotional, and linguistic development, reflected in the various domains of language^(4)^. Typically, the family is the first context with which the child interacts, and it is the family that introduces the first learning and social relationships that are essential for child development. Relationships with parents, siblings, and other family members undergo various transitions throughout life, resulting in the establishment of boundaries and distancing between members, and the roles between and among subsystems are constantly being redefined^(5)^. Thus, the behaviour of one of the members and its consequences are not isolated but influence the entire family system. In other words, the way the family functions affect an individual’s identity and development^(4)^. Longitudinal studies show that children’s resilience is directly related to family support, as they cushion tensions when facing challenges and establish cooperative relationships between them over time. Evidence also shows that children thrive in family structures that are stable, stimulating, and protective^(4)^.

The Circumplex Model was developed to better understand family dynamics and functioning. This model is made up of four key concepts that serve to classify families: cohesion, flexibility, communication, and satisfaction^(6)^. Cohesion is characterised by the emotional bond that family members establish with each other, and it captures how systems balance separation and connection. This dimension is determined by emotional connections, boundaries, alliances, time, space, friends, decision-making, interests, and entertainment. Olson^(6)^ argues that there are four levels of cohesion: disengaged (very low), separated (low to moderate), connected (moderate to high), and enmeshed (very high). Good family functioning is associated with the two intermediate levels (separated and connected), and extremes (disengaged and enmeshed) are usually seen as problematic for long-term family relationships^(6)^.

Flexibility is the family’s capacity for change in leadership, roles, and rules. It refers to how family systems balance stability and change. This dimension is defined by four levels: rigid (very low), structured (low to moderate), flexible (moderate to high), and chaotic (very high)^(6)^. As with cohesion, good family functioning is associated with the two intermediate levels (structured and flexible), and the extremes (rigid and chaotic) are seen as problematic for families throughout their life cycle^(7)^. Communication is seen as the facilitating dimension of the Circumplex Model. This critical dimension helps families change their levels of cohesion and flexibility. Olson^(6)^ indicated that balanced family systems tend to maintain good communication, while unbalanced family systems tend to have poorer communication^(6)^. Lastly, satisfaction refers to the degree of happiness felt by family members^(6)^.

To measure family functioning, based on the Circumplex Model, the *Family Adaptability and Cohesion Evaluation Scale IV* (FACES-IV) was developed to assess the four dimensions of the model mentioned above^(6,8)^. By analysing these characteristics, families can be classified into six types: balanced, rigidly balanced, rigidly cohesive, average midrange, flexibly unbalanced, chaotically disengaged, and unbalanced^(7)^. Balanced families are the healthiest type of family. These families tend to react well to stressful situations and changes in family relationships^(6)^. Rigidly cohesive families have high levels of closeness and rigidity. This type of family is considered to function well due to the high level of closeness between its members but can present difficulties in making the changes necessary for development due to their high levels of rigidity^(7)^. Midrange families tend to function adequately, as they are not at the extreme levels of the dimensions analysed^(6)^. Flexibly unbalanced families appear to have problematic functioning from the outset, but high levels of flexibility provide the necessary capacity to modify problematic dimensions if necessary^(7)^. Chaotically disengaged families are considered problematic due to their lack of emotional closeness and low levels of flexibility, which lead to difficulty in promoting change^(7)^. Unbalanced families are the most problematic type of family in terms of family functioning due to the great lack of emotional closeness and difficulty in adapting^(7)^. Balanced levels of cohesion, flexibility, communication, and satisfaction are associated with a more balanced and functional family.

Family functioning, understood as a process in which family members interact with each other to meet basic needs, make decisions, establish rules, and set goals, simultaneously contributes to individual and family development^(9)^. The introduction of electronic devices into the family context can change family dynamics and lead to adaptations. The role that technology plays in the lives of families and the time spent using it depend on multiple variables, such as socio-economic status, geographical distance between family members, communication strategies established within the family, cultural differences, the fulfilment of needs, and the stage the family is at in its life cycle^(9)^.

Some studies have highlighted a positive role for the use of digital devices as facilitators of communication and coordination of activities between family members, but others have pointed to the negative effects of these devices on family relationships and family cohesion^(10)^. McDaniel^(11)^ stated that family interactions are shaped by the technologies present in the home and that although these can bring families closer together, they can also become a barrier to parent-child interaction time. Several studies have shown an association between excessive parental use of mobile phones in the presence of children and a lower quality of parenting (for example, greater parental permissiveness and more impulsiveness)^(11)^.

Some studies have suggested that children’s learning can be conditioned in various areas by the use of digital devices. Recent research^(9)^ has concluded that watching videos on digital devices can lead to the acquisition of new vocabulary by the children who watch them^(9)^. Nevertheless, interaction is known to play a fundamental role in language development. A child may be exposed to language through screens, but if they do not interact with or use language in their daily lives to express themselves, they will not be able to acquire or use the language^(12)^.

As underscored in European and American studies, exposure to screens varies between 1 and 3 hours a day for children between the ages of 2 and 5. However, this consumption can be moderated by family members. Parental guidance consists of various measures by which parents mould and regulate their children’s use of digital devices^(10)^. Parents can set limits on the amount of device usage, the content consumed, and the contexts in which this consumption takes place^(11)^. The parental use of digital devices is itself indicative of the amount of time their children spend using these devices. A study conducted in 2015 showed that parents who are heavy users of technology are less prohibitive of their children’s use of digital devices^(13)^.

Therefore, this research aims to analyse the relationship between the use of digital devices, family functioning, and language development in preschool children, exploring the associations that these variables may have with each other. At a time when the use of digital devices, such as smartphones or tablets, has grown exponentially, this study arises from the need to obtain more information about their impact on child development and to understand how family functioning can influence this use.

## METHODS

### Study design

A cross-sectional, observational, descriptive-correlational study was carried out.

### Characterisation of the sample

A non-probabilistic convenience sample was selected with the following inclusion criteria: the children had to be aged between 3 years and 0 months and 5 years and 11 months and have European Portuguese as their mother tongue. All children diagnosed with a language disorder associated with a biomedical condition were excluded. The selection of the parental figures followed the inclusion criterion of European Portuguese as a native language, and the exclusion criterion was a diagnosis of severe cognitive and/or psychiatric disorder.

The data on the sample’s sociodemographic characterisation are shown in Table 1, which describes the families and children participating in the study. Initially, a sample of 102 participants was recruited. However, after the children were assessed and the parents filled in the questionnaires, it was realised that 9 of the children had a language disorder associated with a biomedical condition. These participants were therefore excluded from the study, and the final total sample of children consisted of 93 participants. The average age of the children was 57.01 ± 9.95 months, and the majority were female (n = 54; 58.1%).

**Table 1:** Sociodemographic characterisation of the families in the sample

	**n = 93 (100%)**
**Family member's gender**	
**Female (mum)**	81 (87.1%)
**Male (father)**	12 (12.9%)
**Educational qualifications**	
**Up to primary school (4th grade)**	1 (1.1%)
**Up to the 2nd cycle of basic education (6th grade)**	3 (3.2%)
**Up to the 3rd cycle of basic education (9th grade)**	13 (14.0%)
**Up to secondary education (12th grade)**	29 (31.2%)
**Higher education**	47 (50.5%)
**Marital status**	
**Single**	14 (15.1%)
**Married**	45 (48.4%)
**Separate**	3 (3.2%)
**Divorced**	8 (8.6%)
**Widowed**	1 (1.1%)
**Common-law marriage**	22 (23.7%)
**Habitual Occupation**	
**Paid work**	79 (84.9%)
**Unemployed (other reasons)**	8 (8.6%)
**Housework**	4 (4.3%)
**Student**	2 (2.2%)
**Number of children**	
**One**	34 (36.6%)
**Two**	44 (47.3%)
**Three or more**	15 (16.1%)
**Family Structure**	
**Two parents (biological)**	73 (78.5%)
**Only one parent**	12 (12.9%)
**A biological father and a stepfather**	8 (8.6%)

### Data collection procedures

This study was approved by the Ethics Committee of the Health Sciences Research Unit: Nursing (UICISA: E) (Approval no. 697_07-2020). A request for authorisation was sent by email to 30 private institutions of social solidarity (IPSS) and private preschool institutions to ask for their cooperation in the study. Nine educational institutions responded to the invitation (7 IPSS and 2 private schools). The children’s parents were provided with information about the purpose of the study, its objectives, and procedures via email and through documentation sent to them in paper format by the kindergarten teachers. Voluntary participation was requested, and after clarification and agreement to take part, the participants were asked to sign an informed consent form. Data collection took place between October 2021 and March 2022, during which time the data collection protocol was applied to each participating family-child dyad.

A data collection protocol was drawn up that included: (i) a questionnaire to collect sociodemographic and clinical information, (ii) the Portuguese version of the Family Flexibility and Cohesion Evaluation Scale - Version IV (FACES-IV), (iii) a questionnaire on the use of digital devices, and (iv) the Preschool Language Test (Teste de Linguagem - Avaliação da Linguagem Pré-Escolar, TL-ALPE)^(14)^. The children were assessed using the TL-ALPE in person by two speech therapists, and the parents answered the self-completion questionnaires independently.

### Data collection instruments

The FACES-IV is the most recent and complete version of a group of instruments developed to assess family functioning^(6)^. It is a self-completion and family self-assessment instrument based on the Circumplex Model of marital and family systems proposed by Olson^(15)^. The FACES-IV consists of 62 items, divided into 8 subscales: 2 balanced scales (cohesion and flexibility), 4 unbalanced scales (disengaged, enmeshed, rigid, and chaotic), communication, and satisfaction. Each item corresponds to a Likert scale with five response options. In the items assessing the balanced and unbalanced scales, the response options are: (1) Strongly disagree, (2) Disagree, (3) Neither agree nor disagree, (4) Agree, and (5) Strongly agree. In the items assessing communication and satisfaction, the options are: (1) Very dissatisfied, (2) Dissatisfied, (3) Generally satisfied, (4) Very satisfied, and (5) Totally satisfied^(6,7)^. The final scores for each family are calculated by adding up all the items and assigning a final score to each sub-scale. These scores can be converted into qualitative levels, organised by ordered score ranges^(6,7)^.

Regarding psychometric characteristics, the FACES-IV shows robust Cronbach’s alphas, revealing good internal consistency (0.89 in the cohesion subscale, 0.84 in the flexibility subscale, 0.87 in the disengaged subscale, 0.77 in the enmeshed subscale, 0.82 in the rigid subscale, and 0.86 in the chaotic subscale)^(6,7)^. The translation and use of the FACES-IV in this study were duly authorised by the author of the original instrument.

The questionnaire on the use of digital devices (see Appendix 1) in preschool children was developed as part of this study and aimed to help identify the participants’ habits of using these devices, using questions relating to the type of devices most frequently used and the time and moments of the day when use occurred. The questionnaire asked the participants questions assessing their use of electronic devices. In Questions 1, 2a, and 2b, a 5-point Likert scale was used to identify the time spent using each device (1, never; 2, 0-1 hours a day; 3, 1-3 hours a day; 4, 3-6 hours a day; and 5, more than 6 hours a day). Question 3 targeted an understanding of the circumstances under which the children used electronic devices, presenting two answer options: alone or accompanied by an adult. In Questions 4a and 4b, parents were asked to reflect on a possible increase in the use of these devices, influenced by the COVID-19 pandemic, thus presenting two response possibilities (yes - increased; no - not increased).

The TL-ALPE assesses listening comprehension (C) and oral verbal expression (OVE) skills (in the semantic and morphosyntactic domains) in children aged 3-5 years and 12 months^(14)^. The instrument consists of a picture book, a record sheet, and a set of objects of its own that stimulate the children’s responses according to the assessor’s instructions^(14)^. This test is administered individually to each child and should be carried out in a communication-friendly environment with few visual stimuli. Each item has a model, assigned by the assessor, and specific stimuli to which the child responds. This instrument consists of 19 subtests. The correspondence between the score obtained by the child and the standardised OVE and C scores obtained in the TL-ALPE is provided. In the standardised score, a value of 100 corresponds to the mean of the sub-sample with a standard deviation of 15, that is, a value below 100 indicates a result below the mean, and a value above 100 indicates a result above the mean. In this study, the averages of the standardised scores obtained by the children assessed are presented.

### Statistical analysis

The data collected were entered and analysed using the Statistical Package for Social Sciences (SPSS - version 28), using descriptive statistics to calculate absolute and relative frequencies, means, and standard deviations (SD). Inferential analyses were also carried out, calculating non-parametric tests, such as Spearman’s correlation coefficient, since the sample did not follow a normal distribution, as observed by the Shapiro-Wilk normality test. For these calculations, a *p*-value of less than 0.05 was considered statistically significant.

The following values were used to interpret the results and the strength of the correlations: 0.00 to 0.30 (0.00 to - 0.30) weak positive/negative correlation; 0.40 to 0.60 (-0.40 to -0.60) moderate positive/negative correlation; 0.70 to 0.90 (-0.70 to -0.90) strong positive/negative correlation; and 1 (-1) perfect positive/negative correlation^(16)^.

## RESULTS

### Descriptive analysis

The FACES-IV was used to obtain data characterising family functioning. These data are shown in Table 2. These data show that most of the families participating in this study were of the balanced type. The data obtained from the questionnaire on the use of digital devices concerned the use of these devices by parents and children. We observed that the devices most used by parents outside working hours were smartphones and television. Of these devices, most parents used them between 0 and 3 hours a day. Most parents (n = 51; 54.8%) also considered that the COVID-19 pandemic had increased the time they used these devices. Further, the devices most used by the children during the week were the smartphone and television. For the most used devices, most participants used them between 0 and 3 hours a day for their smartphones (n = 61; 65.6%) and television (n = 83; 89.2%). When the same question was asked about the period of use during the weekend, the results fluctuated slightly, with a greater difference in the use of the tablet*,* which was used between 1 and 6 hours a day by 33.4% (n = 31) of the children.

**Table 2:** FACES-IV results - Balanced, unbalanced, communication and satisfaction subscales

Scales	Sub-scales	Level	n	%	M ± SD
Balanced	Cohesion	Very connected	68	73.1%	30.17 ± 3.59
		On	23	24.7%	
		Separated	2	2.2%	
	Flexibility	Very flexible	46	49.5%	28.43 ± 3.47
		Flexible	46	49.5%	
		Structured	1	1.1%	
Unbalanced	Disengaged	Moderate	2	2.2%	13.13 ± 3.25
		Bass	10	10.8%	
		Very low	81	87.1%	
	Enmeshed	High	1	1.1%	17.12 ± 3.26
		Moderate	9	9.7%	
		Bass	46	49.5%	
		Very low	37	39.8%	
	Rigid	High	8	8.6%	20.32 ± 3.78
		Moderate	28	30.1%	
		Bass	40	43.0%	
		Very low	17	18.3%	
	Chaotic	Moderate	2	2.2%	14.59 ± 3.34
		Bass	19	20.4%	
		Very low	72	77.4%	
Communication		Very high	36	38.7%	40.63 ± 5.60
		High	35	37.6%	
		Moderate	16	17.2%	
		Bass	3	3.2%	
		Very low	3	3.2%	
Satisfaction		Very high	17	18.3%	38.15 ± 6.30
		High	20	21.5%	
		Moderate	21	22.6%	
		Bass	27	29.0%	
		Very low	8	8.6%	

Regarding the circumstances in which they used digital devices, most participants said that their children used these devices accompanied by an adult (n = 68; 73.1%). Further, most parents indicated that there had been an increase in the time their children used these devices since the emergence of the COVID-19 pandemic (n = 65; 69.9%). The responses also showed that the time of day when each device was most used was after preschool, with proportions of positive responses ranging from 29% to 46.2% for the most-used devices (smartphone, tablet, and television).

The analysis of the TL-ALPE yielded standardised scores for OVE and C. In the OVE, we obtained an average of 98.09 ± 16.8 (with minimum scores of 55 and maximum scores of 131), and in the C, we obtained an average of 97.41 ± 19.56 (with minimum scores of 51 and maximum scores of 127). Thus, the average results obtained on the OVE and C suggested that most of the children assessed had normal language development, although there were 14 children (15.05%) who had more than 1.5 SD below the mean (a cut-off point suggestive of a language disorder) in listening comprehension, and 10 children (10.75%) had more than 1.5 SD below the mean on the OVE.

### Inferential analysis

Tables 3, 4, and 5 show only the statistically significant results of the correlation analyses carried out between the variables analysed. We observed a positive and statistically significant association between the results obtained in the OVE subtests and the FACES-IV cohesion and satisfaction subscales, suggesting that greater family cohesion and satisfaction were associated with a better score in the OVE tests. Regarding the disengaged subscale, there was a statistically significant negative correlation—that is, families with higher levels of disengagement were associated with worse scores on the OVE tests.

**Table 3:** Statistically significant correlations between the dimensions of the TL-ALPE, the FACES-IV and the average time spent using digital devices

TL-ALPE	FACES-IV and the use of digital devices	Spearman's Correlation Coefficient	p
OVE's standardised quotation	Cohesion	0.344	0.001
	Disengaged	- 0.245	0.018
	Satisfaction	0.310	0.002
Standardised C quotation	Cohesion	0.279	0.007
	Disengaged	-0.222	0.032
OVE's standardised quotation	Smartphone use during the week	-0.274	0.008
	Tablet use during the week	-0.343	0.001
	Smartphone use during the weekend	-0.259	0.012
Standardised C quotation	Smartphone use during the week	-0.245	0.018
	Tablet use during the week	-0.331	0.001
	Smartphone use during the weekend	-0.242	0.020
	Computer use during the weekend	-0.230	0.027

**Table 4:** Correlation of results between FACES-IV and average time spent using digital devices

FACES - IV	Use of digital devices	Spearman's Correlation Coefficient	p
Cohesion	Smartphone use during the weekend	-0.205	0.049
Flexibility	Smartphone use during the week	-0.263	0.011
	Smartphone use during the weekend	-0.242	0.020
Enmeshed	Smartphone use during the weekend	0.214	0.039
	Television use at the weekend	0.361	0.000
Chaotic	Television use during the week	0.210	0.043
	Television use at the weekend	0.223	0.031
Satisfaction	Tablet use during the week	-0.205	0.048
	Smartphone use during the weekend	-0.282	0.006

**Table 5:** Correlation of results between the average time parents and children use digital devices during the week and at the weekend

Use of digital devices (parents)	Use of digital devices during the week and at the weekend (children)	Spearman's Correlation Coefficient	p
Smartphone	Smartphone use (week)	0.322	0.002
	Television use (week)	0.236	0.023
Television	Smartphone use (week)	0.315	0.002
	Tablet use (week)	0.241	0.020
	Television use (week)	0.437	0.000
			
Smartphone	Smartphone use (weekend)	0.338	0.001
	Television use (weekend)	0.246	0.018
Tablet	Tablet use (weekend)	0.359	0.000
Television	Smartphone use (weekend)	0.278	0.007
	Television use (weekend)	0.345	0.001

Regarding the C tests, there were statistically significant correlations between this variable and the FACES-IV cohesion and disengagement subscales. Thus, children in families who score higher on cohesion tend to show more favourable results on the C tests, while children whose families show higher levels of disengagement tend to score lower on the same tests (see Table 3). There were statistically significant correlations between OVE and C scores and the time spent using the smartphone, tablet, and computer. The results indicated that the longer each of these devices was used during the week and/or at the weekend, the lower the scores on the OVE and C tests (Table 3).

There were negative and statistically significant correlations between the cohesion, flexibility, and satisfaction scores and the amount of time the children used the smartphone during the week and at the weekend or the amount of time the children used the tablet during the week. As the scores on these subscales of the FACES-IV decreased (i.e. lower cohesion, lower flexibility, and lower satisfaction), the amount of time the children used their smartphone and/or tablet increased. Positive and statistically significant associations were also found between the enmeshed and chaotic subscales and the duration of digital device use in terms of television use during the week and smartphone and television use during the weekend. Thus, higher scores on the enmeshed and chaotic dimensions were associated with more time spent using these devices by the children (Table 4).

Positive and statistically significant correlations were observed between the amount of time parents used certain digital devices and the amount of time children used these devices during the week and on the weekend. The results indicated that more time spent using the smartphone by parents outside of working hours was associated with more hours spent using the smartphone and television by children, both during the week and on the weekend. Greater use of the tablet by parents was also associated with greater use of the same device by children on weekends. Parents’ television consumption times were associated with children’s smartphone and television use times (during the week and at the weekend) and tablet use times (during the week).

## DISCUSSION

This study aimed to analyse the relationship between the use of digital devices, family functioning and language development in preschool children. Although the link between the variables analysed is visible in this study, it is important to point out that, in general, the children assessed showed results that indicate normal language development. Therefore, the associations between the variables should be interpreted carefully, considering one limitation of the study, essentially related to the homogeneity of the sample, which does not allow us to identify more contrasting differences between the elements of the population studied. Furthermore, the strength of the correlations found varies between weak and moderate. It is also important to emphasise that the questionnaire on the use of digital devices was constructed within the scope of this study because, as far as we know, there is no instrument for this purpose validated for the Portuguese population. It would therefore be important to carry out validity and reliability studies on this type of instrument in the future.

Regarding family functioning, most families scored high on the balanced subscales and low on the unbalanced subscales of the FACES-IV, corresponding to a balanced family typology^(7)^. The fact that most of the families were made up of both biological parents and had paid jobs is indicative of a greater family balance. However, families in which both parents have a paid job may indicate less time available for family time and stimulating activities with the children, which can lead to greater use of digital devices that do not require the constant presence of parents^(17)^.

As for the use of digital devices, the participants indicated that the ones most used by their children were smartphones, tablets, and television, with average periods of use between 1 and 3 hours a day. These results are in line with previous studies carried out in European countries and the United States^(18)^, which showed that children aged between 2 and 5 spend between 2 and 3 hours a day using digital devices^(18)^. In the present study, the parents also indicated that the times of day when these devices were most used were “after preschool” and “in the evening, before going to bed”. These periods of increased use may be associated with parents managing household chores, making digital devices a more independent form of occupation for children, and freeing carers to carry out household and family tasks.

Analysis of the scores obtained on the TL-ALPE and the results of the FACES-IV revealed that children with more cohesive family functioning scored better on the C and OVE tests. Similarly, greater family satisfaction was also reflected in higher scores on the OVE tests. However, the C and OVE scores worsened with increased levels of family disengagement. According to the Circumplex Model, more balanced levels of cohesion are associated with good family functioning^(7,19)^. More cohesive families tend to react well to stressful situations and changes in family relationships and tend to be more functional over the course of the family’s life cycle^(7,20)^.

Satisfaction refers to the degree of happiness felt by family members. According to Rebelo^(20)^ families with higher levels of satisfaction have significantly better family communication than families with lower levels of satisfaction. In fact, the data obtained in this study suggest that higher levels of family satisfaction promote good language development at the OVE level^(19,20)^.

Regarding the unbalanced subscales, although the families in this study had average results showing very low levels of disengagement, we found that in more disengaged families, the children scored lower on OVE and C. More disengaged families usually have greater emotional separation. This implies less involvement between family members, where each individual tends to opt for more isolated activities, and members tend not to seek each other out for support and help in solving problems^(15)^. Evidence has shown that language development is influenced by environmental factors present in children’s environments, such as the family environment. Family time spent doing stimulating activities (such as reading a book, using educational games, and talking to the child) plays a fundamental role in the acquisition and development of language skills^(2)^. This may help to understand the results of this study, which suggests that children from families with higher levels of disengagement scored lower on the TL-ALPE tests.

In relation to the TL-ALPE and the use of digital devices, some statistically significant results were observed. We found that children who used smartphones and tablets for longer periods of time had lower scores in terms of language development, suggesting that exposure to screens can compromise language development. Recent studies^(12)^ show that delayed language development may be related to increased screen time^(12)^. This delayed development has been attributed to the replacement of activities that enhance language development (such as playing, painting, and interactive games with adults) with the use of digital devices^(17,21)^. This may help to understand the trends in the results obtained in this study.

However, several authors^(17,22)^ argue that watching videos on digital devices can lead to the acquisition of new vocabulary and that educational television programmes for children can be a useful and economical learning tool^(17,22)^. Interaction with peers or adults (parents, family members, and educators) is known to play a fundamental role in language development. It is therefore essential to train and empower families to mediate the use of this type of device to increase their digital literacy, so that the use of technology can have a more positive impact on language development, enhancing it^(12)^.

Previous studies^(23)^ suggest that parental use of digital devices is related to and influences their children’s use of the same devices^(23)^. In the present study, we found that parental use of smartphones, tablets, and television was associated with longer periods of use of the same devices by children. One way children learn is through observation, and this form of learning has practical implications for parental use of technology in the presence of children, as it can lead to the modelling of observed behaviour^(23)^.

Smartphones were identified in this study as the most used device, both by parents and children, corroborating the results of previous studies^(24-28)^. Smartphones present themselves as particularly challenging devices in terms of imposing limits and rules of use^(23)^. Therefore, parents’ ability to regulate their children’s use is closely related to their digital literacy. In short, the use of digital devices (especially smartphones) can have a positive or negative impact on children’s development, depending on how this use is mediated by parents^(27)^.

With the emergence of the COVID-19 pandemic in 2020, several countries resorted to lockdown measures as a way of containing this infection, leading to long periods of isolation for families in their homes. In this study, participants indicated an increase in the use of digital devices by both parents (54.8%) and children (69.9%) during this period. As has already been discussed, such an increase leads to a decrease in children’s interactions with peers and other adults, thus limiting the number of stimuli and their quality for their language development^(29)^.

In this study, negative and statistically significant correlations were observed between the cohesion, flexibility, and satisfaction subscales and smartphone use (during the week and at the weekend) and tablet use (during the week). This indicates that as the time spent using these devices increased, the levels of cohesion, flexibility, and family satisfaction decreased. Therefore, in families with lower levels of cohesion, flexibility, and family satisfaction, the children spent more time using digital devices. In addition, in two unbalanced subscales (tangled and chaotic), there was a positive relationship between these and *smartphone* use (during the weekend) and television use (during the week and during the weekend). This suggests that the time spent using devices is greater in families with higher levels of enmeshment and chaos.

A recent study^(10)^ indicated that frequent use of digital devices can negatively influence family cohesion and flexibility, with healthier family functioning when the child’s daily digital use is lower^(10)^. Another study, conducted in 2017^(29)^ showed that less use of digital devices is related to families with stronger emotional bonds, emotional reciprocity, and mutual respect between parents and children, which are fundamental characteristics for greater family cohesion^(29)^. Further, more flexible families are better able to deal with change and adapt to and learn from different family situations, leading to practical implications for leadership processes, discipline, and rules^(10)^. The use of digital devices can reduce face-to-face relationships between family members and increase stressful family situations. Therefore, greater use of digital devices can coincide with less family flexibility^(29)^.

Thus, the data from this study have significant implications for health and education professionals who deal with monitoring child development. By offering new perspectives on the impact of family functioning and the use of digital devices on children’s language, this study provides important information to guide interventions and strategies aimed at improving children’s language development. An in-depth understanding of these factors can help create environments that are more conducive to learning and semantic and syntactic enrichment, directly benefiting language development and preparing children for future challenges. However, it is essential to emphasise that further research with larger and more heterogeneous samples is needed to fully understand the complexity of these relationships. Thus, this study can be a starting point for future research, providing contributions to furthering knowledge about children’s language development and the impact of family functioning and the use of digital devices.

## CONCLUSION

This study concluded that children with more cohesive family functioning and good levels of family satisfaction scored higher on the oral verbal expression (OVE) and comprehension (C) tests of the TL-ALPE and that the scores of children with greater family disengagement were lower on these tests. Further, the use of some digital devices (smartphone, tablet, and computer) was significantly associated with the scores obtained in the OVE and C tests, and the number of hours spent using these devices also affected the children’s scores on the language assessment tests, with more hours spent using digital devices being associated with lower scores. Finally, an increase in the time spent using digital devices by preschool children was associated with lower levels of family cohesion, flexibility, and satisfaction and higher levels of family enmeshment and chaos.

In conclusion, this study highlights the impact of the use of digital devices and the role of family functioning in preschool children’s language development. The results show that excessive use of digital devices can be associated with less balanced dimensions of family functioning and jeopardise comprehensive language development. In this sense, it is considered that moderate use of digital devices (up to a maximum of 1 hour per day for children up to the age of 5, according to the World Health Organisation^(30)^) and a healthy and engaging family environment are fundamental to promoting satisfactory language development.

## Appendix 1 Questionnaire on the use of digital devices in pre-school children

This questionnaire aims to analyse the use of digital devices by the family.

Read each question carefully and put a cross (x) in the option or options that best correspond to your perception of what happens in your family.

There are no "right" or "wrong" answers. What really counts is your perspective on the use of electronic devices in the family.

Thank you so much!

1- How much time a day does the parent use each of the following digital devices (outside of working time)?

**Table:**

**1**	**2**	**3**	**4**	**5**
Never	0-1 hours per day	1-3 hours a day	3-6 hours a day	More than 6 hours a day

**Table:**

Mobile phone/Smartphone	□ 1 □ 2 □ 3 □ 4 □ 5
Tablet	□ 1 □ 2 □ 3 □ 4 □ 5
Computer/Laptop/Notebook	□ 1 □ 2 □ 3 □ 4 □ 5
Television	□ 1 □ 2 □ 3 □ 4 □ 5
Radio	□ 1 □ 2 □ 3 □ 4 □ 5
Video games	□ 1 □ 2 □ 3 □ 4 □ 5

2- How much time a day do you allow your child to use each of the following digital devices?

a. During the week.

**Table:**

**1**	**2**	**3**	**4**	**5**
Never	0-1 hours per day	1-3 hours a day	3-6 hours a day	More than 6 hours a day

**Table:**

Mobile phone/Smartphone	□ 1 □ 2 □ 3 □ 4 □ 5
Tablet	□ 1 □ 2 □ 3 □ 4 □ 5
Computer/Laptop/Notebook	□ 1 □ 2 □ 3 □ 4 □ 5
Television	□ 1 □ 2 □ 3 □ 4 □ 5
Radio	□ 1 □ 2 □ 3 □ 4 □ 5
Video games	□ 1 □ 2 □ 3 □ 4 □ 5

b. During the weekend.

**Table:**

**1**	**2**	**3**	**4**	**5**
Never	0-1 hours per day	1-3 hours a day	3-6 hours a day	More than 6 hours a day

**Table:**

Mobile phone/Smartphone	□ 1 □ 2 □ 3 □ 4 □ 5
Tablet	□ 1 □ 2 □ 3 □ 4 □ 5
Computer/Laptop/Notebook	□ 1 □ 2 □ 3 □ 4 □ 5
Television	□ 1 □ 2 □ 3 □ 4 □ 5
Radio	□ 1 □ 2 □ 3 □ 4 □ 5
Video games	□ 1 □ 2 □ 3 □ 4 □ 5

3- In general, under what circumstances does your child use digital devices?

□ Alone □ Accompanied by an adult

4- Do you consider that the COVID-19 pandemic has increased the usage time of those devices by:

a. Your child?

□ Yes □ No

b. Mum/dad?

□ Yes □ No

Thank you for your co-operation!
