# Supplementary material for: Use of digital devices, family functioning, and language development in preschool children: a cross-sectional study
Source: Codas. 2024 Mar 18;36(3):e20230125. doi: 10.1590/2317-1782/20232023125en (PMC10984584; doi:10.1590/2317-1782/20232023125en)
Supplement: Translation

# Translation: Utilização de dispositivos digitais, funcionamento familiar e desenvolvimento da linguagem em crianças de idade pré-escolar: um estudo transversal

**DOI:** 10.1590/2317-1782/20232023125pt

**Authors:** Maria Inês Figueiras Gomes, Marisa Lobo Lousada, Daniela Maria Pias de Figueiredo

**Affiliations:** 1 Escola Superior de Saúde, Universidade de Aveiro - ESSUA, Aveiro, Portugal.; 2 Centro de Investigação em Tecnologias e Serviços e Saúde - CINTESIS, Universidade de Aveiro, Aveiro, Portugal.

**Keywords:** Desenvolvimento da Linguagem, Desenvolvimento Infantil, Idade Pré-escolar, Tecnologia Digital, Dinâmica Familiar

## Abstract

**Objetivo:**

Este estudo teve como objetivo analisar a relação entre a utilização de dispositivos digitais, o funcionamento familiar e o desenvolvimento da linguagem em crianças de idade pré-escolar.

**Método:**

Estudo transversal, descritivo-correlacional com uma amostra de 93 díades pais-crianças. As crianças tinham uma média etária de 57,01 ± 9,95 meses, sendo a maioria do gênero feminino. Os instrumentos de recolha de dados incluíram um questionário sobre o uso de dispositivos digitais, a versão portuguesa da Escala de Avaliação da Flexibilidade e Coesão Familiar - Versão IV (FACES-IV) e o Teste de Linguagem - Avaliação de Linguagem Pré-Escolar (TL-ALPE).

**Resultados:**

As respostas demonstram uma maior tendência para a utilização do smartphone, tablet e televisão entre 0 e 3 horas por dia nas crianças. Com a aplicação da FACES-IV e do TL-ALPE, verificou-se que a maioria das famílias participantes eram do tipo equilibrado e que a maioria das crianças apresenta um normal desenvolvimento da linguagem. Observaram-se relações estatisticamente significativas entre a FACES-IV e o TL-ALPE; a FACES-IV e a utilização de dispositivos digitais; a utilização de dispositivos digitais e o TL-ALPE. Verificou-se que crianças com um funcionamento familiar mais equilibrado pontuam mais alto nas provas do TL-ALPE e que o tempo de uso de dispositivos digitais pode comprometer o desenvolvimento da linguagem.

**Conclusão:**

Destaca-se o impacto da utilização dos dispositivos digitais e o papel do funcionamento familiar no desenvolvimento da linguagem da criança, sugerindo que uma utilização moderada de dispositivos digitais e um funcionamento familiar equilibrado são fatores facilitadores de um bom desenvolvimento da linguagem.

## INTRODUÇÃO

Um adequado desenvolvimento da linguagem é um dos principais fatores para que o desenvolvimento infantil ocorra de forma harmoniosa em todos os seus domínios, quer do ponto de vista social, relacional ou de aprendizagem formal. Trata-se de um processo progressivo, diferencial e específico de cada criança^(1,2)^.

A linguagem é adquirida de forma natural e espontânea e manifesta-se pela aquisição de uma língua. Para que tal aconteça, a criança tem de estar exposta a essa língua^(3)^. É possível identificar no desenvolvimento da linguagem uma ordem sequencial de aquisições e marcos de desenvolvimento que ocorrem aproximadamente na mesma idade, em todas as crianças. No entanto, ao longo do desenvolvimento da linguagem, existem diferenças individuais, tanto no que respeita ao processo de aquisição como à qualidade e rapidez da mesma^(3)^.

Na medida em que as crianças adquirem a língua usada na comunidade em que crescem, o respectivo comportamento linguístico reflete as diferenças e especificidades do sistema e do meio envolvente^(2)^. Consequentemente, o desenvolvimento da linguagem depende não somente das condições biológicas inatas de cada indivíduo, como também sofre influências de fatores ambientais presentes nos meios em que as crianças estão inseridas, como por exemplo, a família ou a escola^(4)^.

Relativamente às condições biológicas, quanto aos fatores genéticos, alguns autores indicam que um historial de perturbações da linguagem na família influencia diretamente a criança, logo é comum que crianças com uma perturbação da linguagem tenham pelo menos um familiar que também teve uma alteração similar na infância. Estudos também indicam que as perturbações da linguagem são mais prevalentes nas crianças do sexo masculino, sugerindo o sexo da criança é também um fator genético relevante^(4)^.

Quanto aos fatores ambientais, o meio em que a criança se insere é fundamental para a aquisição e desenvolvimento da linguagem, uma vez que sem essa influência, as competências genéticas para a aquisição da linguagem não serão desenvolvidas. O meio assume, assim, um papel crucial no processo de aquisição e desenvolvimento da linguagem, pois nele a criança vai desenvolver-se de forma progressiva e natural pela exposição à língua da comunidade onde se insere, de acordo com o seu ritmo individual de desenvolvimento^(4)^.

A literatura sugere a existência de fatores que influenciam o desenvolvimento da linguagem e dos estilos educativos parentais adotados, tais como o nível socioeconómico familiar, a escolaridade dos pais, presença ou ausência de irmãos, entre outros^(4)^.

Meios mais estimulantes exercem uma influência essencial na organização cerebral, proporcionando experiências de interação mais ricas que possibilitam o desenvolvimento cognitivo, funcional, social, emocional e linguístico, refletindo-se nos vários domínios da linguagem^(4)^.

Tipicamente, a família é o primeiro contexto com o qual a criança interage, sendo esta que introduz as primeiras aprendizagens e relações sociais que são essenciais para o desenvolvimento infantil. Os relacionamentos com pais, irmãos e outros elementos familiares, passam por várias transições ao longo da vida, o que implica que os limites estabelecidos, a distância entre os membros e os papéis dentre e entre subsistemas estejam em constante redefinição^(5)^. Assim, o comportamento de um dos membros e as suas consequências não são isoladas, influenciando todo o sistema familiar. Ou seja, o funcionamento da família tem efeitos na identidade de um indivíduo e no seu desenvolvimento^(4)^.

Estudos longitudinais referem que a resiliência das crianças está diretamente relacionada com o apoio familiar, pois diminuem as tensões ao enfrentar desafios, estabelecendo relações cooperativas entre eles, ao longo do tempo. Há ainda estudos que mencionam que as crianças podem prosperar em estruturas familiares que são estáveis, estimuladoras e protetoras^(4)^.

Com o objetivo de melhor compreender a dinâmica e o funcionamento familiar foi desenvolvido o modelo circumplexo. Este modelo é composto por quatro conceitos-chave que servem para a posterior classificação das famílias: coesão, flexibilidade, comunicação e satisfação^(6)^.

A coesão é caracterizada pelo vínculo emocional que os membros da família estabelecem entre si e o seu objetivo é compreender de que forma os sistemas equilibram a separação e a ligação. Esta dimensão é determinada por ligações emocionais, limites, alianças, tempo, espaço, amigos, tomadas de decisão, interesses e entretenimento. Olson^(6)^ defende a existência de quatro níveis de coesão: desligada (muito baixa), separada (baixa a moderada), ligada (moderada a alta), emaranhada (muito alta). O bom funcionamento familiar é associado aos dois níveis intermédios (separada e ligada) e os extremos (desligada e emaranhada) são normalmente vistos como problemáticos para relacionamentos familiares a longo prazo^(6)^.

A flexibilidade é a capacidade familiar de mudança na liderança, papéis e regras. Foca-se na forma como os sistemas familiares equilibram a estabilidade e a mudança. Esta dimensão é definida por quatro níveis de flexibilidade: rígida (muito baixa), estruturada (baixa a moderada), flexível (moderada a alta), caótica (muito alta)^(6)^. Tal como na coesão, o bom funcionamento familiar está associado aos dois níveis intermédios (estruturada e flexível) e os extremos (rígida e caótica) são vistos como problemáticos para as famílias ao longo do seu ciclo de vida^(7)^.

A comunicação é vista como a dimensão facilitadora do Modelo Circumplexo. É assim considerada como uma dimensão crítica que auxilia as famílias a alterar os seus níveis de coesão e flexibilidade. Olson^(6)^ indica que sistemas familiares equilibrados tendem a manter uma boa comunicação, enquanto sistemas familiares desequilibrados tendem a ter uma comunicação mais pobre^(6)^.

Por fim, a satisfação diz respeito ao grau de felicidade sentido pelos membros da família^(6)^.

De modo a medir o funcionamento familiar, tendo por base o Modelo Circumplexo, foi desenvolvida a *Family Adaptability and Cohesion Evaluation Scale IV* (FACES-IV) para avaliar as quatro dimensões do modelo anteriormente mencionadas^(6,8)^. Por meio da análise destas características as famílias podem ser classificadas em 6 tipos: equilibrada, rigidamente equilibrada, média, flexivelmente desequilibrada, caoticamente desligada e desequilibrada^(7)^.

As famílias equilibradas são consideradas como sendo o tipo de família mais saudável. Estas famílias tendem a reagir bem perante situações de stress e alterações nas relações familiares^(6)^.

As famílias rigidamente equilibradas apresentam níveis elevados de proximidade e de rigidez. Considera-se que este tipo de famílias funciona bem devido ao elevado nível de proximidade entre os seus elementos, porém, podem apresentar dificuldades em realizar as mudanças necessárias para o desenvolvimento, dado os seus níveis elevados de rigidez^(7)^.

As famílias médias, apresentam, tendencialmente, um funcionamento adequado, pois não se situam nos níveis extremos das dimensões em análise^(6)^.

As famílias flexivelmente desequilibradas aparentam ter, a princípio, um funcionamento problemático, no entanto, os níveis elevados de flexibilidade proporcionam as capacidades necessárias para modificar as dimensões problemáticas caso seja necessário^(7)^.

As famílias caoticamente desligadas são consideradas famílias problemáticas devido à falta de proximidade emocional e aos baixos níveis de flexibilidade, que levam à dificuldade em promover mudanças^(7)^.

As famílias desequilibradas são consideradas como o tipo de família mais problemático ao nível do funcionamento familiar devido à grande falta de proximidade emocional e dificuldade de adaptação^(7)^.

Verifica-se então que níveis equilibrados de coesão, flexibilidade, comunicação e satisfação estão associados a um funcionamento familiar mais equilibrado e funcional.

O funcionamento familiar, sendo compreendido como um processo em que os membros da família interagem uns com os outros para corresponder às necessidades básicas, tomar decisões, estabelecer regras e definir objetivos, contribui, simultaneamente, para o desenvolvimento individual e familiar^(9)^.

A introdução de dispositivos eletrónicos no contexto familiar pode alterar as dinâmicas familiares, levando a adaptações. O papel que as tecnologias assumem na vida das famílias e o tempo gasto na sua utilização é dependente de múltiplas variáveis, tais como: o nível socioeconómico, a distância geográfica entre membros da família, as estratégias comunicativas estabelecidas pelo seio familiar, as diferenças culturais, a satisfação de necessidades e o estádio em que a família se encontra no seu ciclo vital^(9)^.

Alguns estudos evidenciam que existe um papel positivo na utilização de dispositivos digitais como facilitadores da comunicação e da coordenação de atividades entre os membros da família, mas outros apontam para os efeitos negativos destes dispositivos nas relações familiares e na coesão das famílias^(10)^.

McDaniel^(11)^ afirma que as interações familiares são moldadas pelas tecnologias presentes em casa e que, apesar destas poderem aproximar as famílias, também podem tornar-se numa barreira em relação ao tempo de interação pais-criança. Vários estudos ao longo dos anos têm demonstrado uma associação entre a utilização parental excessiva de telemóveis na presença dos filhos e uma menor qualidade do exercício parental (por exemplo, maior permissividade parental e mais impulsividade)^(11)^.

Nos últimos anos, tem surgido alguma literatura que sugere que as aprendizagens das crianças podem ser condicionadas, em vários domínios, pela utilização de dispositivos digitais. Pesquisas recentes^(9)^ concluíram que a visualização de vídeos em dispositivos digitais pode levar à aquisição de novo vocabulário por parte das crianças que os veem^(9)^. Apesar disto, sabe-se que a interação tem um papel fundamental no desenvolvimento da linguagem. A criança pode estar exposta à linguagem devido as telas, mas, não havendo interação nem utilização da linguagem no dia a dia por parte da mesma para se expressar, a criança não será capaz de adquirir e usar de forma adequada a linguagem a que foi exposta^(12)^.

Tal como é mencionado em estudos europeus e americanos, a exposição a telas varia entre uma a três horas por dia, para crianças entre os dois e os cinco anos de idade. No entanto, este consumo pode ser moderado pelos familiares. A orientação parental consiste em várias medidas por meio das quais os pais moldam e regulam a utilização dos dispositivos digitais por parte das crianças^(10)^. Os pais podem estabelecer limites na quantidade de utilização dos dispositivos, nos conteúdos consumidos e nos contextos em que estes consumos ocorrem^(11)^. A própria utilização parental dos dispositivos digitais é um indicativo do tempo que os seus filhos passam a utilizar esses dispositivos. Um estudo conduzido em 2015, mostrou que os pais que são grandes utilizadores de tecnologias são menos proibitivos relativamente à utilização de dispositivos digitais por parte dos seus filhos^(13)^.

Portanto, a presente investigação tem como objetivo analisar a relação entre a utilização de dispositivos digitais, o funcionamento familiar e o desenvolvimento da linguagem em crianças de idade pré-escolar, explorando as associações que estas variáveis podem ter entre elas. Numa altura em que a utilização dos dispositivos digitais, como *smartphones* ou *tablets*, tem crescido de forma exponencial, este estudo surge da necessidade em obter mais informação sobre o impacto dos mesmos no desenvolvimento infantil e perceber de que modo o funcionamento familiar pode influenciar essa utilização.

## MÉTODO

### Desenho de estudo

Foi desenvolvido um estudo com um desenho transversal, observacional, descritivo-correlacional.

### Caracterização da amostra

Foi selecionada uma amostra não probabilística e por conveniência, com os seguintes critérios de inclusão: as crianças tinham de ter idades compreendidas entre os 3 anos e 0 meses e os 5 anos e 11 meses, e ter o Português Europeu como língua materna. Foram excluídas todas as crianças que apresentassem diagnóstico de perturbação de linguagem associada a uma condição biomédica.

Quanto às figuras parentais, definiu-se como critério de inclusão ter o Português Europeu como língua materna e como critério de exclusão a presença de diagnóstico de perturbação cognitiva e/ou psiquiátrica grave.

Os dados relativos à caracterização sociodemográfica da amostra são apresentados na Tabela 1, referindo-se às famílias e às crianças participantes no estudo, respetivamente. Inicialmente, foi recolhida uma amostra de 102 participantes. No entanto, após a avaliação das crianças e preenchimento dos questionários por parte dos pais verificou-se que 9 das crianças participantes apresentavam uma perturbação da linguagem associada a uma condição biomédica. Assim, esses participantes foram excluídos do estudo, sendo a amostra total final de crianças constituída por 93 participantes.

**Tabela 1:** Caracterização sociodemográfica das famílias da amostra

	**n = 93 (100%)**
**Género do familiar**	
**Feminino (mãe)**	81 (87,1%)
**Masculino (pai)**	12 (12,9%)
**Habilitações Literárias**	
**Até ao 1º ciclo do ensino básico (4º ano)**	1 (1,1%)
**Até ao 2º ciclo do ensino básico (6º ano)**	3 (3,2%)
**Até ao 3º ciclo do ensino básico (9º ano)**	13 (14,0%)
**Até ao ensino secundário (12º ano)**	29 (31,2%)
**Ensino superior**	47 (50,5%)
**Estado Civil**	
**Solteiro**	14 (15,1%)
**Casado**	45 (48,4%)
**Separado**	3 (3,2%)
**Divorciado**	8 (8,6%)
**Viúvo**	1 (1,1%)
**União de fato**	22 (23,7%)
**Ocupação Habitual**	
**Trabalho remunerado**	79 (84,9%)
**Desempregado (outros motivos)**	8 (8,6%)
**Trabalho doméstico**	4 (4,3%)
**Estudante**	2 (2,2%)
**Número de filhos**	
**Um**	34 (36,6%)
**Dois**	44 (47,3%)
**Três ou mais**	15 (16,1%)
**Estrutura da Família**	
**Dois pais (biológicos)**	73 (78,5%)
**Apenas um dos pais**	12 (12,9%)
**Um pai biológico e um padrasto**	8 (8,6%)

No que diz respeito às crianças, a média etária foi de 57,01 ± 9,95 meses e a maioria é do género feminino (n=54; 58,1%).

### Procedimentos de recolha de dados

Este estudo obteve parecer favorável da Comissão de Ética da Unidade de Investigação em Ciências da Saúde: Enfermagem (UICISA: E) (Parecer nº 697_07-2020).

Foi realizado um pedido de autorização via email a 30 Instituições Particulares de Solidariedade Social (IPSS) e colégios privados com pré-escola, de forma a solicitar a sua colaboração no estudo. Obteve-se aceitação de 9 instituições educacionais (7 IPSS e 2 colégios particulares).

Foi facultada aos pais das crianças, através de documentação enviada aos mesmos em formato papel por intermédio dos educadores de infância, informação acerca do propósito do estudo, os seus objetivos e procedimentos via email. Solicitou-se a participação voluntária e após os esclarecimentos e concordância em participar, os participantes foram convidados a assinar o Termo de Consentimento Livre e Esclarecido. A recolha de dados decorreu entre outubro de 2021 e março de 2022, período em que foi aplicado o protocolo de recolha de dados a cada díade família-criança participantes.

Foi elaborado um protocolo de recolha de dados que incluiu: i) um questionário para recolha de informação sociodemográfica e clínica; ii) a versão portuguesa da Escala de Avaliação da Flexibilidade e Coesão Familiar - Versão IV (FACES-IV); iii) um questionário sobre a utilização dos dispositivos digitais; iv) e o Teste de Linguagem - Avaliação de Linguagem Pré-Escolar (TL-ALPE)^(14)^. As crianças foram avaliadas com recurso ao TL-ALPE presencialmente por duas Terapeutas da Fala e os pais responderam autonomamente aos questionários de autopreenchimento.

### Instrumentos de recolha de dados

A FACES-IV é a versão mais recente e completa de um grupo de instrumentos desenvolvidos com o objetivo de avaliar o funcionamento familiar^(6)^. É um instrumento de autopreenchimento e autoavaliação familiar que se apoia no Modelo Circumplexo dos sistemas conjugal e familiar, proposto por Olson^(15)^. A FACES-IV é composta por 62 itens, divididos em oito subescalas: duas escalas equilibradas (coesão e flexibilidade); quatro escalas desequilibradas (desligamento, emaranhada, rígida e caótica); comunicação e satisfação. A cada item corresponde uma escala de Likert com cinco opções de resposta. Nos itens de avaliação das escalas equilibradas e desequilibradas, as opções de resposta são: 1) Discordo fortemente, 2) Discordo, 3) Não concordo nem discordo, 4) Concordo e 5) Concordo fortemente. Nos itens de avaliação da comunicação e da satisfação, as opções são: 1) Muito insatisfeito, 2) Insatisfeito, 3) Geralmente satisfeito, 4) Muito satisfeito e 5) Totalmente satisfeito (6,7).

As pontuações finais de cada família são calculadas através da soma de todos os itens que atribui uma cotação final a cada subescala. Estas cotações podem ser convertidas em níveis qualitativos, organizados por intervalos ordenados de pontuações^(6,7)^.

Relativamente às características psicométricas, a FACES-IV apresenta valores de alfas de Cronbach robustos, revelando uma boa consistência interna (0,89 na subescala de coesão; 0,84 na subescala de flexibilidade; 0,87 na subescala de desligamento; 0,77 na subescala emaranhada; 0,82 na subescala de rigidez; 0,86 na subescala caótica^(6,7)^. A tradução e uso da FACES-IV no âmbito deste estudo foi devidamente autorizada pelo autor do instrumento original.

O questionário sobre a utilização de dispositivos digitais (ver Apêndice 1) em crianças em idade pré-escolar foi desenvolvido no âmbito deste estudo e visa auxiliar na identificação dos hábitos de utilização destes aparelhos por parte dos participantes, recorrendo a perguntas relativas ao tipo de aparelhos mais frequentemente utilizados, tempo e momentos do dia em que ocorre a utilização.

Através deste questionário foi solicitada aos participantes a resposta às questões apresentadas avaliando a utilização dos dispositivos eletrónicos. Nas perguntas 1, 2a e 2b, utilizou-se uma escala de tipo Likert de 5 pontos para identificar o tempo passado a utilizar cada um dos dispositivos (1- nunca; 2- 0-1 horas por dia; 3- 1-3 horas por dia; 4- 3-6 horas por dia; 5- mais de 6 horas por dia). Na pergunta 3, com o objetivo de perceber em que circunstâncias as crianças utilizam os dispositivos eletrónicos, foram apresentadas duas opções de resposta (sozinho(a); acompanhado(a) por um adulto). Nas questões 4a e 4b, solicitou-se aos pais a reflexão sobre um possível aumento da utilização desses dispositivos, influenciado pela pandemia por COVID-19, apresentando assim duas possibilidades de resposta (sim - aumentou; não - não aumentou).

O Teste de Linguagem - Avaliação de Linguagem Pré-Escolar (TL-ALPE) avalia as competências de compreensão auditiva (CA) e expressão verbal oral (EVO) (nos domínios semântico e morfossintático) em crianças dos 3 aos 5 anos e 12 meses^(14)^.

O instrumento é constituído por um livro de imagens, uma folha de registo e um conjunto de objetos próprios que estimulam as respostas das crianças de acordo com as instruções do avaliador^(14)^.

A aplicação deste teste é feita individualmente a cada criança e deve ser realizada num ambiente facilitador à comunicação e com poucos estímulos visuais. Cada item tem um modelo, dado pelo avaliador, e estímulos específicos para obtenção da resposta por parte da criança. Este instrumento é constituído por dezanove subtestes. É disponibilizada a correspondência entre a cotação obtida pela criança com os valores da cotação estandardizada da EVO e da CA obtidos no TL-ALPE. Na cotação estandardizada, um valor de 100 corresponde à média da subamostra com um desvio padrão de 15, ou seja, um valor inferior a 100 indica um resultado abaixo da média e um valor superior a 100 indica um resultado acima da média. Neste estudo, são apresentadas as médias das cotações estandardizadas obtidas pelas crianças avaliadas.

### Análise estatística

Os dados recolhidos foram introduzidos e analisados no programa de análise estatística *Statistical Package for Social Sciences* (SPSS - versão 28), com recurso à estatística descritiva, através do cálculo de frequências absolutas, relativas, médias e desvios-padrão. Também se realizaram análises inferenciais, com o cálculo de testes não paramétricos como o coeficiente de correlação de Spearman, pelo fato de a amostra não seguir uma distribuição normal, tal como observado pelo teste de normalidade de Shapiro-Wilk. Para estes cálculos, um valor de *p* inferior a 0,05 foi considerado estatisticamente significativo.

Para interpretar os resultados e a força das correlações, consideraram-se os seguintes valores: 0.00 a 0.30 (0.00 a - 0.30) correlação positiva/negativa fraca; 0.40 a 0.60 (-0.40 a -0.60) correlação positiva/negativa moderada; 0.70 a 0.90 (-0.70 a -0.90) correlação positiva/negativa forte; 1 (-1) correlação positiva/negativa perfeita^(16)^.

## RESULTADOS

### Análise descritiva

Com a aplicação da FACES-IV obtiveram-se dados que caracterizam o funcionamento familiar. Estes dados são apresentados na Tabela 2. Com estes dados, verificou-se que a maioria das famílias participantes neste estudo é do tipo equilibrado.

**Tabela 2:** Resultados da FACES-IV - Subescalas equilibradas, desequilibradas, de comunicação e de satisfação

Escalas	Subescalas	Nível	n	%	M ± DP
Equilibradas	Coesão	Muito ligada	68	73,1%	30,17 ± 3,59
		Ligada	23	24,7%	
		Separada	2	2,2%	
	Flexibilidade	Muito flexível	46	49,5%	28,43 ± 3,47
		Flexível	46	49,5%	
		Estruturada	1	1,1%	
Desequilibradas	Desligamento	Moderado	2	2,2%	13,13 ± 3,25
		Baixo	10	10,8%	
		Muito baixo	81	87,1%	
	Emaranhada	Alto	1	1,1%	17,12 ± 3,26
		Moderado	9	9,7%	
		Baixo	46	49,5%	
		Muito baixo	37	39,8%	
	Rigidez	Alto	8	8,6%	20,32 ± 3,78
		Moderado	28	30,1%	
		Baixo	40	43,0%	
		Muito baixo	17	18,3%	
	Caótica	Moderado	2	2,2%	14,59 ± 3,34
		Baixo	19	20,4%	
		Muito baixo	72	77,4%	
Comunicação		Muito alto	36	38,7%	40,63 ± 5,60
		Alto	35	37,6%	
		Moderado	16	17,2%	
		Baixo	3	3,2%	
		Muito baixo	3	3,2%	
Satisfação		Muito alto	17	18,3%	38,15 ± 6,30
		Alto	20	21,5%	
		Moderado	21	22,6%	
		Baixo	27	29,0%	
		Muito baixo	8	8,6%	

Os dados obtidos no questionário sobre a utilização dos dispositivos digitais dizem respeito à utilização dos mesmos por parte dos pais e das crianças.

Observou-se que os dispositivos mais utilizados pelos pais fora do horário laboral são o *smartphone* e a televisão. Desses dispositivos, o tempo de utilização da maioria dos pais varia entre 0 e 3 horas por dia. A maioria dos pais (n=51; 54,8%) considerou ainda que a pandemia por COVID-19 aumentou o tempo de utilização desses dispositivos.

Observou-se também que os dispositivos mais utilizados pelas crianças durante a semana são o *smartphone* e a televisão. Dos dispositivos mais utilizados, o tempo de utilização da maioria dos participantes varia entre 0 e 3 horas por dia para o *smartphone* (n=61; 65,6%) e para a televisão (n=83; 89,2%). Quando a mesma questão é colocada relativamente ao período de utilização durante o fim de semana, os resultados oscilam ligeiramente, notando-se uma maior diferença na utilização do *tablet* que passa a ser utilizado por 33,4% (n=31) das crianças entre 1 e 6 horas por dia.

Relativamente às circunstâncias de utilização dos dispositivos digitais, a maioria dos participantes afirma que os filhos utilizam estes dispositivos acompanhados por um adulto (n=68; 73,1%). De igual modo, a maioria dos pais indica ter havido um aumento do tempo de utilização destes dispositivos por parte dos filhos com o surgimento da pandemia por COVID-19 (n=65; 69,9%).

Com a aplicação deste questionário, observou-se ainda que o período do dia em que cada um dos dispositivos é mais utilizado é depois da pré-escola, com percentagens de respostas positivas que variam entre 29% e 46,2%, para os dispositivos mais utilizados (*smartphone*, *tablet* e televisão).

Com a aplicação do TL-ALPE, foi possível obter as cotações estandardizadas na EVO e na CA. Na EVO obteve-se uma média de 98,09 ±16,8 (com cotações mínimas de 55 e máximas de 131) e na CA obteve-se uma média de 97,41 ± 19,56 (com cotações mínimas de 51 e máximas de 127). Assim, os resultados médios obtidos na EVO e na CA sugerem que a maioria das crianças avaliadas apresentam um normal desenvolvimento da linguagem, havendo, no entanto, 14 crianças (15.05%) que têm mais do que 1,5 de desvio padrão abaixo da média (ponto de corte sugestivo de perturbação de linguagem) na compreensão auditiva e 10 crianças (10.75%) têm mais do que 1,5 de desvio padrão abaixo da média na EVO.

### Análise inferencial

As Tabelas 3, 4 e 5 apresentam apenas os resultados estatisticamente significativos das análises de correlação efetuadas entre as variáveis analisadas.

**Tabela 3:** Correlações estatisticamente significativas entre as dimensões do TL-ALPE, da FACES-IV e o tempo médio de utilização dos dispositivos digitais

TL-ALPE	FACES-IV e utilização dos dispositivos digitais	Coeficiente de Correlação de Spearman	p
Cotação estandardizada da EVO	Coesão	0,344	0,001
	Desligamento	- 0,245	0,018
	Satisfação	0,310	0,002
Cotação estandardizada da CA	Coesão	0,279	0,007
	Desligamento	-0,222	0,032
Cotação estandardizada da EVO	Utilização do *smartphone* durante a semana	-0,274	0,008
	Utilização do *tablet* durante a semana	-0,343	0,001
	Utilização do *smartphone* durante o fim de semana	-0,259	0,012
Cotação estandardizada da CA	Utilização do *smartphone* durante a semana	-0,245	0,018
	Utilização do *tablet* durante a semana	-0,331	0,001
	Utilização do *smartphone* durante o fim de semana	-0,242	0,020
	Utilização do computador durante o fim de semana	-0,230	0,027

**Tabela 4:** Correlação de resultados entre a FACES-IV e o tempo médio de utilização de dispositivos digitais

FACES - IV	Utilização dos dispositivos digitais	Coeficiente de Correlação de Spearman	p
Coesão	Utilização do *smartphone* durante o fim de semana	-0,205	0,049
Flexibilidade	Utilização do *smartphone* durante a semana	-0,263	0,011
	Utilização do *smartphone* durante o fim de semana	-0,242	0,020
Emaranhada	Utilização do *smartphone* durante o fim de semana	0,214	0,039
	Utilização da televisão durante o fim de semana	0,361	0,000
Caótica	Utilização da televisão durante a semana	0,210	0,043
	Utilização da televisão durante o fim de semana	0,223	0,031
Satisfação	Utilização do *tablet* durante a semana	-0,205	0,048
	Utilização do *smartphone* durante o fim de semana	-0,282	0,006

**Tabela 5:** Correlação de resultados entre o tempo médio de utilização de dispositivos digitais dos pais e das crianças durante a semana e durante o fim de semana

Utilização dos dispositivos digitais (pais)	Utilização dos dispositivos digitais durante a semana e durante o fim de semana (crianças)	Coeficiente de Correlação de Spearman	p
Utilização do *smartphone*	Utilização do *smartphone* (semana)	0,322	0,002
	Utilização da televisão (semana)	0,236	0,023
Utilização da televisão	Utilização do *smartphone* (semana)	0,315	0,002
	Utilização do *tablet* (semana)	0,241	0,020
	Utilização da televisão (semana)	0,437	0,000
			
Utilização do *smartphone*	Utilização do *smartphone* (fim de semana)	0,338	0,001
	Utilização da televisão (fim de semana)	0,246	0,018
Utilização do *tablet*	Utilização do *tablet* (fim de semana)	0,359	0,000
Utilização da televisão	Utilização do *smartphone* (fim de semana)	0,278	0,007
	Utilização da televisão (fim de semana)	0,345	0,001

Verificou-se que, nos resultados obtidos nos subtestes de expressão verbal oral (EVO), existe uma associação positiva e estatisticamente significativa entre estes e as subescalas Coesão e Satisfação da FACES-IV, sugerindo que a maior coesão e satisfação familiar se associa uma melhor pontuação nas provas de EVO. Relativamente à subescala de desligamento, verificou-se a existência de uma correlação negativa estatisticamente significativa, ou seja, em famílias com níveis mais elevados de desligamento associa-se uma pior pontuação nas provas de EVO.

Quanto aos resultados obtidos nas provas de compreensão auditiva (CA), verificaram-se correlações estatisticamente significativas entre esta variável e as subescalas Coesão e Desligamento da FACES-IV. Assim, as crianças em famílias que pontuam mais alto na coesão tendem a evidenciar resultados mais favoráveis nas provas de CA enquanto as crianças cujas famílias evidenciam níveis mais elevados de desligamento, tendem a pontuar mais baixo nas mesmas provas (ver Tabela 3).

Observaram-se correlações estatisticamente significativas entre as pontuações da EVO e CA e o tempo de utilização do *smartphone*, *tablet* e computador. Os resultados obtidos indicam que, quanto maior é o tempo de utilização de cada um destes dispositivos durante a semana e/ou ao fim de semana, mais baixas são as pontuações nas provas de EVO e CA (ver Tabela 3).

Observaram-se correlações negativas e estatisticamente significativas entre as pontuações da Coesão, Flexibilidade e Satisfação e o tempo de utilização do *smartphone* durante a semana e durante o fim de semana e a utilização do *tablet* durante a semana por parte da criança. Ou seja, à medida que diminuem as pontuações nestas subescalas da FACES-IV (i.e., menor coesão, menor flexibilidade e menor satisfação), aumenta o tempo de utilização do *smartphone* e/ou do *tablet* das crianças. Foram também encontradas associações positivas e estatisticamente significativas entre as subescalas Emaranhada e Caótica e a duração da utilização de dispositivos digitais referentes à utilização da televisão durante a semana e do *smartphone* e da televisão durante o fim de semana. Assim, pontuações mais elevadas nas dimensões emaranhada e caótica associam-se a mais tempo de utilização destes dispositivos por parte das crianças (ver Tabela 4).

Observaram-se correlações positivas e estatisticamente significativas entre o tempo de utilização de alguns dispositivos digitais por parte dos pais e o tempo de utilização desses dispositivos por parte das crianças, durante a semana e durante o fim de semana. Os resultados indicam que, a mais tempo de utilização do *smartphone* por parte dos pais, fora do horário laboral, se associa mais horas despendidas na utilização do *smartphone* e da televisão, por parte das crianças, tanto durante a semana como durante o fim de semana. A maior utilização do *tablet* por parte dos pais, está também associada a uma maior utilização do mesmo dispositivo pelas crianças, durante o fim de semana. Os tempos de consumo da televisão por parte dos pais associam-se com os tempos de utilização, nas crianças, do *smartphone* e da televisão (durante a semana e durante o fim de semana) e do *tablet* (durante a semana).

## DISCUSSÃO

Este estudo teve como objetivo analisar a relação entre a utilização de dispositivos digitais, o funcionamento familiar e o desenvolvimento da linguagem em crianças de idade pré-escolar.

Apesar de, no presente estudo, ser visível a ligação entre as variáveis analisadas torna-se importante ressalvar que, na generalidade, as crianças avaliadas apresentam resultados que indicam um normal desenvolvimento da linguagem. Assim, as associações verificadas entres as variáveis devem ser cautelosamente interpretadas tendo em consideração as limitações do estudo, que se prendem, essencialmente, com a homogeneidade da amostra, não permitindo identificar diferenças mais contrastantes entre os elementos da população estudada. Para além disso, a força das correlações encontradas varia entre fraca e moderada. É também importante ressalvar que o questionário sobre a utilização dos dispositivos digitais foi construído no âmbito deste estudo pois, tanto quanto se sabe, não existe qualquer instrumento com este propósito validado para a população portuguesa. Assim, seria relevante futuramente realizarem-se estudos de validade e fiabilidade deste tipo de instrumentos.

Relativamente ao funcionamento familiar, verifica-se que a maioria das famílias pontua valores altos nas subescalas equilibradas e valores baixos nas subescalas desequilibradas da FACES-IV, correspondendo a uma tipologia familiar equilibrada^(7)^.

O fato de a maior parte das famílias ser constituída pelos dois pais biológicos e ter um trabalho remunerado, são fatores indicativos de um maior equilíbrio familiar. Por outro lado, famílias em que os dois progenitores têm um trabalho remunerado pode implicar menos tempo disponível para passar em família e para realizar atividades estimulantes com as crianças, o que pode levar a uma maior utilização de dispositivos digitais que não implique a presença constante dos pais^(17)^.

Assim, quanto à utilização dos dispositivos digitais, os participantes indicaram que os mais utilizados pelos seus filhos são o *smartphone*, o *tablet* e a televisão, com períodos de utilização médios situados entre 1 e 3 horas por dia. Estes resultados vão ao encontro de estudos previamente realizados em países Europeus e nos Estados Unidos^(18)^, que mostraram que crianças entre os 2 e os 5 anos passam entre 2 e 3 horas por dia a utilizar dispositivos digitais^(18)^. No presente estudo, foi também indicado pelos pais que as alturas do dia em que estes dispositivos são mais utilizados são “depois da pré-escola” e “à noite, antes de deitar”. Estes períodos de maior utilização podem estar associados aos momentos de gestão de tarefas domésticas por parte dos pais, tornando-se os dispositivos digitais uma forma de ocupação mais independente para as crianças, libertando os cuidadores para a realização das tarefas domésticas e familiares.

Analisando as pontuações obtidas no TL-ALPE e os resultados da FACES-IV, verificou-se que crianças com um funcionamento familiar mais coeso pontuam melhor nas provas de CA e EVO. De igual modo, uma maior satisfação familiar também se reflete em pontuações mais elevadas nas provas de EVO. Por outro lado, as pontuações ao nível da CA e da EVO pioram quando os níveis de desligamento familiar aumentam. De acordo com o Modelo Circumplexo, níveis mais equilibrados de coesão estão associados a um bom funcionamento familiar^(7,19)^. As famílias mais coesas, tendem a reagir bem perante situações de stress e alterações nas relações familiares e tendem a ser mais funcionais no decorrer do ciclo vital da família^(7,20)^.

A satisfação diz respeito ao grau de felicidade sentido pelos membros da família. Segundo Rebelo^(20)^, famílias com níveis mais elevados de satisfação terão uma comunicação familiar significativamente melhor do que as famílias com níveis mais baixos de satisfação. De fato, os dados obtidos no presente estudo sugerem que níveis mais elevados de satisfação familiar promovem um bom desenvolvimento da linguagem, ao nível da EVO^(19,20)^.

Além disso, relativamente às subescalas desequilibradas, embora as famílias do presente estudo tenham apresentado resultados médios reveladores de níveis muito baixos de desligamento, verificou-se ainda assim que em famílias mais desligadas as crianças pontuam mais baixo na EVO e na CA. As famílias mais desligadas apresentam, normalmente, uma maior separação emocional. Isto implica um menor envolvimento entre os membros da família, onde cada indivíduo tende a optar por atividades mais isoladas e os membros tendem a não procurarem uns pelos outros para obter apoio e auxílio na resolução de problemas^(15)^. A evidência tem demonstrado que o desenvolvimento da linguagem é influenciado pelos fatores ambientais presentes nos meios em que as crianças se inserem, como é o caso do seio familiar. O tempo passado em família a realizar atividades estimulantes (como ler um livro, utilizar jogos didáticos, ter momentos de conversação com a criança), tem um papel fundamental na aquisição e desenvolvimento das competências linguísticas^(2)^. Este fato pode ajudar a compreender os resultados do presente estudo que sugerem que crianças inseridas em meios familiares com níveis de desligamento mais elevados apresentem pontuações mais baixas nas provas do TL-ALPE.

Em relação ao TL-ALPE e à utilização dos dispositivos digitais, foram observados alguns resultados com significância estatística. Verifica-se que em crianças com um maior tempo de utilização de *smartphones* e *tablets*, as pontuações em termos de desenvolvimento da linguagem são inferiores, sugerindo que o tempo de exposição a telas pode comprometer o desenvolvimento da linguagem. Estudos recentes^(12)^ demonstram que o desenvolvimento tardio da linguagem pode estar relacionado com o aumento do tempo de exposição a telas^(12)^. Este desenvolvimento tardio tem sido atribuído à substituição de atividades que potenciam o desenvolvimento da linguagem (tais como brincar, pintar, realizar jogos interativos com o adulto) pela utilização de dispositivos digitais^(17,21)^, o que pode ajudar a compreender a tendência dos resultados obtidos no presente estudo.

No entanto, vários autores^(17,22)^ defendem que a visualização de vídeos em dispositivos digitais pode levar à aquisição de novo vocabulário e que programas de televisão educativos para crianças podem ser uma ferramenta útil e económica de aprendizagem^(17,22)^. Sabe-se que a interação com os pares ou com adultos (pais, familiares e educadores) têm um papel fundamental no desenvolvimento da linguagem. Assim, torna-se essencial formar e capacitar as famílias para a mediação da utilização deste tipo de dispositivos, aumentando a sua literacia digital, para que a utilização da tecnologia possa ter um impacto mais positivo no desenvolvimento da linguagem, potenciando-o^(12)^.

Estudos anteriores^(23)^ sugerem que a utilização parental de dispositivos digitais está relacionada e influencia a utilização dos seus filhos dos mesmos dispositivos^(23)^. No presente estudo, verificou-se que a utilização parental de *smartphones*, *tablets* e televisão está associada a maiores períodos de utilização dos mesmos dispositivos por parte das crianças. Sabe-se que uma das formas de aprendizagem das crianças ocorre através da observação. Esta forma de aprendizagem tem implicações práticas relativamente à utilização parental da tecnologia na presença das crianças, pois pode levar a uma modelagem do comportamento observado^(23)^.

Os *smartphones* foram identificados neste estudo como sendo o dispositivo mais utilizado, quer por pais como por crianças, corroborando os resultados já evidenciados por estudos prévios^(24-28)^. Os *smartphones* apresentam-se como um dispositivo particularmente desafiante no que toca à imposição de limites e regras de utilização^(23)^, pelo que a capacidade dos pais para regularem o seu uso por parte dos filhos está intimamente relacionada com a sua literacia digital. Em suma, a utilização de dispositivos digitais (em especial, do *smartphone*) pode ter impactos positivos ou negativos no desenvolvimento infantil dependendo da forma como essa utilização é mediada pelos pais^(27)^.

Desde 2020, com o surgimento da pandemia por COVID-19, vários países recorreram a medidas de confinamento como forma de contenção desta infecção levando a longos períodos de isolamento das famílias nas suas habitações. Neste estudo, os participantes indicaram um aumento da utilização dos dispositivos digitais quer por parte dos pais (54,8%), quer por parte das crianças (69,9%). Como já foi discutido, este aumento, leva a uma diminuição das interações das crianças com os pares e com outros adultos, limitando assim o número de estímulos e qualidade dos mesmos^(29)^.

Neste estudo, observaram-se correlações negativas e estatisticamente significativas entre as subescalas de coesão, flexibilidade e satisfação e a utilização do *smartphone* (durante a semana e durante o fim de semana) e do *tablet* (durante a semana). Isto indica que, conforme o tempo de utilização destes dispositivos aumenta, os níveis de coesão, flexibilidade e satisfação familiar diminuem. Logo, em famílias com menores níveis de coesão, flexibilidade e satisfação familiar verifica-se um maior tempo despendido na utilização de dispositivos digitais por parte das crianças. Adicionalmente, em duas subescalas desequilibradas (emaranhada e caótica) verificou-se uma relação positiva entre estas e a utilização do *smartphone* (durante o fim de semana) e da televisão (durante a semana e durante o fim de semana). Isto sugere que o tempo de utilização dos dispositivos é maior em famílias cujos níveis de emaranhamento e de caos também é mais elevado.

Um estudo recente^(10)^ indicou que a utilização frequente de dispositivos digitais pode influenciar negativamente a coesão e a flexibilidade familiar, havendo um funcionamento familiar mais saudável quando a utilização digital diária da criança é menor^(10)^. Outro estudo, conduzido em 2017^(29)^, evidenciou que uma menor utilização de dispositivos digitais se relaciona com famílias com laços emocionais mais fortes, reciprocidade emocional e respeito mútuo entre pais e filhos, características fundamentais para uma maior coesão familiar^(29)^. Sabe-se ainda que famílias mais flexíveis conseguem lidar de forma mais adequada com a mudança, adaptar-se e aprender com as diversas situações familiares, levando a implicações práticas nos processos de liderança, disciplina e regras^(10)^. A utilização de dispositivos digitais pode diminuir as relações cara-a-cara entre os membros da família e aumentar situações familiares estressantes. Logo, uma maior utilização de dispositivos digitais pode ser coincidente com uma menor flexibilidade familiar^(29)^.

Assim, os dados do presente estudo têm implicações significativas para os profissionais de saúde e educação que lidam com o acompanhamento do desenvolvimento infantil. Ao oferecer novas perspectivas sobre o impacto do funcionamento familiar e do uso de dispositivos digitais na linguagem infantil, este estudo pode fornecer informações importantes para orientar intervenções e estratégias voltadas para o aprimoramento do desenvolvimento linguístico das crianças. O entendimento aprofundado desses fatores pode contribuir para a criação de ambientes mais propícios à aprendizagem e enriquecimento semântico e sintático, beneficiando diretamente o desenvolvimento linguístico e a preparação das crianças para os desafios futuros. No entanto, é fundamental ressaltar que pesquisas adicionais com amostras mais alargadas e heterogêneas são necessárias para compreender plenamente a complexidade dessas relações. Assim, este estudo pode ser um ponto de partida para investigações futuras, fornecendo contributos para o aprofundamento dos conhecimentos sobre o desenvolvimento linguístico infantil e o impacto que o funcionamento familiar e a utilização de dispositivos digitais exercem sobre o mesmo.

## CONCLUSÃO

Este estudo permitiu concluir que crianças com um funcionamento familiar mais coeso e com bons níveis de satisfação familiar pontuam mais alto nas provas expressão verbal oral (EVO) e de compreensão auditiva (CA) do TL-ALPE, e ainda que, crianças com um maior desligamento familiar pontuam mais baixo nestas provas. Observou-se também que a utilização de alguns dispositivos digitais (*smartphone*, *tablet* e computador) se associa significativamente com as pontuações obtidas nas provas de EVO e de CA e que, o número de horas despendido na utilização destes dispositivos também condiciona as pontuações obtidas nas provas de avaliação de linguagem, verificando-se que mais horas passadas a utilizar dispositivos digitais se associam a pontuações mais baixas nos testes de avaliação de linguagem. Por fim, verificou-se que a um aumento do tempo de utilização de dispositivos digitais por parte de crianças em idade pré-escolar se associam níveis mais baixos de coesão, flexibilidade e satisfação familiar e níveis mais elevados de emaranhamento e caos familiar.

Concluindo, este estudo destaca o impacto da utilização dos dispositivos digitais e o papel do funcionamento familiar no desenvolvimento da linguagem da criança em idade pré-escolar. Os resultados mostram que uma utilização excessiva de dispositivos digitais pode estar associada a dimensões menos equilibradas de funcionamento familiar e comprometer o desenvolvimento integral da linguagem. Neste sentido, considera-se que uma utilização moderada dos dispositivos digitais (até um máximo de 1 hora por dia para crianças até aos 5 anos de idade, de acordo com a Organização Mundial de Saúde^(30)^) e um ambiente familiar saudável e envolvente, são fundamentais para promover um desenvolvimento linguístico satisfatório.

## Apêndice 1 Questionário sobre a utilização de dispositivos digitais em crianças com idade pré-escolar

Este questionário visa analisar o uso de dispositivos digitais pela família.

Leia atentamente cada questão e coloque uma cruz (x) na ou nas opções que melhor correspondem à perceção relativamente ao que acontece na sua família

Não existem respostas “certas” ou “erradas”. O que conta verdadeiramente é a sua perspetiva em relação à utilização de dispositivos eletrónicos em família.

Muito obrigada!

1- Quanto tempo por dia o/a pai/mãe utiliza cada um dos seguintes dispositivos digitais (fora do tempo laboral)?

**Table:**

**1**	**2**	**3**	**4**	**5**
Nunca	0-1 horas por dia	1-3 horas por dia	3-6 horas por dia	Mais de 6 horas por dia

**Table:**

Telemóvel/Smartphone	□ 1 □ 2 □ 3 □ 4 □ 5
Tablet	□ 1 □ 2 □ 3 □ 4 □ 5
Computador/Laptop/Portátil	□ 1 □ 2 □ 3 □ 4 □ 5
Televisão	□ 1 □ 2 □ 3 □ 4 □ 5
Rádio	□ 1 □ 2 □ 3 □ 4 □ 5
Videojogos	□ 1 □ 2 □ 3 □ 4 □ 5

2- Quanto tempo por dia permite que o(a) seu filho(a) utilize cada um dos seguintes dispositivos digitais?

a. Durante a semana.

**Table:**

**1**	**2**	**3**	**4**	**5**
Nunca	0-1 horas por dia	1-3 horas por dia	3-6 horas por dia	Mais de 6 horas por dia

**Table:**

Telemóvel/Smartphone	□ 1 □ 2 □ 3 □ 4 □ 5
Tablet	□ 1 □ 2 □ 3 □ 4 □ 5
Computador/Laptop/Portátil	□ 1 □ 2 □ 3 □ 4 □ 5
Televisão	□ 1 □ 2 □ 3 □ 4 □ 5
Rádio	□ 1 □ 2 □ 3 □ 4 □ 5
Videojogos	□ 1 □ 2 □ 3 □ 4 □ 5

b. Durante o fim de semana.

**Table:**

**1**	**2**	**3**	**4**	**5**
Nunca	0-1 horas por dia	1-3 horas por dia	3-6 horas por dia	Mais de 6 horas por dia

**Table:**

Telemóvel/Smartphone	□ 1 □ 2 □ 3 □ 4 □ 5
Tablet	□ 1 □ 2 □ 3 □ 4 □ 5
Computador/Laptop/Portátil	□ 1 □ 2 □ 3 □ 4 □ 5
Televisão	□ 1 □ 2 □ 3 □ 4 □ 5
Rádio	□ 1 □ 2 □ 3 □ 4 □ 5
Videojogos	□ 1 □ 2 □ 3 □ 4 □ 5

3- De um modo geral, em que circunstâncias é que o(a) seu filho(a) utiliza os dispositivos digitais:

□ Sozinho(a) □ Acompanhado(a) por um adulto

4- Considera que a pandemia por COVID-19 aumentou o tempo de utilização desses dispositivos por parte:

a. Do(a) seu filho?

□ Sim □ Não

b. Do pai/mãe?

□ Sim □ Não

Obrigada pela sua colaboração!
